# Cancer Biomarkers: Reflection on Recent Progress, Emerging Innovations, and the Clinical Horizon

**DOI:** 10.3390/cancers17182981

**Published:** 2025-09-12

**Authors:** M. Walid Qoronfleh, Nader Al-Dewik

**Affiliations:** 1 Healthcare Research & Policy Division, Q3 Research Institute (QRI), Ypsilanti, MI 48917, USA; 2 Healix Lab, Al Khuwair South, Muscat 123, Oman; 3 AtomGen, Esenşehir Mah. Güneyli Sokak No:15/1, 34776 Ümraniye, İstanbul, Türkiye; 4 Department of Research & Pediatrics, Women’s Wellness and Research Center, Hamad Medical Corporation, Doha 00974, Qatar; 5 Genomics and Precision Medicine (GPM), College of Health & Life Science (CHLS), Hamad Bin Khalifa University (HBKU), Doha 34110, Qatar; 6 Faculty of Health and Social Care Sciences, Kingston University, St. George’s University of London, London KT1 2EE, UK

**Keywords:** biomarkers, cancer, precision medicine, translational medicine, clinical trials, companion diagnostics, early detection, screening, therapy

## Abstract

This perspective provides a short overview of cancer biomarkers, balancing the technical details with the broad implications for biomarker discovery and innovation; early detection and screening; personalized treatment and monitoring; and emerging technologies. It also briefly discusses challenges in their clinical translation while exploring recent advancements and future implications for clinical practice. Finally, we offer thoughts on the role of artificial intelligence (AI) in biomarker development. AI is accelerating the discovery and validation of biomarkers by mining complex datasets, identifying hidden patterns, and improving the predictive accuracy. AI-powered tools enhance image-based diagnostics, automate genomic interpretation, and facilitate real-time monitoring of treatment responses. By integrating multi-omics data, AI offers new avenues for precision medicine and scalable cancer diagnostics, pushing biomarker development into a new era of intelligent, data-driven oncology. This editorial is a reflection on the state of biomarkers based on the contributions to the Special Issue “Cancer Biomarkers: Recent Progress, Innovations, and Future Clinical Implications”.

## 1. Introduction

Cancer biomarkers are biological molecules—such as proteins, genes, or metabolites—that can be objectively measured to indicate the presence, progression, or behavior of cancer. These markers are indispensable in modern oncology, playing pivotal roles in early detection, diagnosis, treatment selection, and monitoring of therapeutic responses. As cancer continues to be a leading cause of mortality worldwide—with an estimated 20 million new cases and 9.7 million deaths in 2022 alone—the development and application of biomarkers have become essential for improving patient outcomes and advancing precision medicine [1]. Over the past decade, remarkable progress in biomarker research, fueled by cutting-edge platform technologies and innovative ‘multi-omics’ approaches, has expanded their clinical utility. This article delves into the current state of cancer biomarkers, highlights recent innovations, and explores their future implications for transforming cancer care.

The importance of biomarkers lies in their ability to provide actionable insights into a disease that is notoriously complex and heterogeneous. From screening asymptomatic populations to tailoring therapies to individual patients, biomarkers are bridging the gap between basic research and clinical practice [2]. Indeed, biomarkers can significantly enhance therapy outcomes, thus saving lives, lessening suffering, and diminishing psychological and economic burdens. This editorial will examine their applications across the cancer care continuum, address the challenges hindering their widespread adoption, and envision a future where biomarkers drive a more proactive and personalized approach to oncology (precision oncology).

## 2. Biomarkers in Cancer Screening and Early Detection

### 2.1. Established Biomarkers and Their Limitations

Screening and monitoring for cancer aim to detect the disease in its earliest stages when treatment is most likely to succeed. Several cancer diagnostics biomarkers are being utilized, including CEA, AFP, CA 19-9, and PSA, as well as emerging biomarkers like circulating tumor cells (CTCs), circulating tumor DNA (ctDNA), cell-free DNA (cfDNA), circulating free RNA (cfRNA), microRNAs (miRNAs), long non-coding RNAs (lncRNAs), DNA methylation and tumor-derived extracellular vesicles (EV) markers [2,3]. Traditional biomarkers, such as prostate-specific antigen (PSA) for prostate cancer and cancer antigen 125 (CA-125) for ovarian cancer, have been widely used for this diagnostic purpose. However, these markers often disappoint due to limitations in their sensitivity and specificity, resulting in overdiagnosis and/or overtreatment in patients. For example, PSA levels can rise due to benign conditions like prostatitis or benign prostatic hyperplasia, leading to false positives and unnecessary invasive procedures. Similarly, CA-125 is not exclusive to ovarian cancer and can be elevated in other cancers or non-malignant conditions, such as endometriosis. Therefore, this necessitates careful interpretation of the results and often requires further investigation. These shortcomings highlight the urgent need for more reliable screening tools. Another common issue is that many established biomarkers do not emerge until the cancer is already advanced, reducing their value in early detection [3].

### 2.2. Recent Progress in Discovering New Screening Biomarkers

Recent advances in the field of omics technologies such as genomics, epigenomics, transcriptomic, proteomics, and metabolomics have accelerated the discovery of novel biomarkers for early detection [2]. Screening for specific mutations in genes like KRAS, EGFR, and TP53 or using oncology gene panels for mutations that drive the development and progression of cancer has been the most direct approach. One standout example is circulating tumor DNA (ctDNA) as a non-invasive biomarker that detects fragments of DNA shed by cancer cells into the bloodstream [3]. ctDNA has shown promise in detecting various cancers—such as lung, breast, and colorectal—at the preclinical stages, offering a window for intervention before symptoms appear. Additionally, multi-analyte blood tests combining DNA mutations, methylation profiles, and protein biomarkers—such as CancerSEEK—have demonstrated the ability to detect multiple cancer types simultaneously, with encouraging sensitivity and specificity [4].

Another advancement is the FDA-approved OVA1 test. While not a screening tool, this multi-marker panel measures the levels of five protein biomarkers in the blood, which aids in risk stratification and facilitates the referral of high-risk women with ovarian cancer [NIH-EDRN, available online: https://edrn.nci.nih.gov/data-and-resources/biomarkers/ova1/, accessed on 20 August 2025]. These breakthroughs are laying the groundwork for early detection screening tools, which could significantly reduce cancer mortality.

### 2.3. Innovations in the Detection Technologies for Early Cancer Detection

Technological innovations are augmenting the precision and accessibility of biomarker detection. Liquid biopsies, which analyze ctDNA or CTCs from a blood sample, are gaining traction and represent a non-invasive alternative to traditional tissue biopsies [3]. This method permits early detection and real-time monitoring of cancers like lung and colorectal cancer, with the added benefit of being less burdensome for patients. Meanwhile, artificial intelligence (AI) and machine learning (ML) are revolutionizing biomarker analysis by identifying subtle patterns or signatures in large datasets that human observers might miss. AI/ML enable the integration and analysis of various molecular data types with imaging to provide a vivid picture of the cancer, consequently enhancing the diagnostic accuracy and recommendations of the appropriate therapies. These and other technologies are improving the accuracy of early detection and paving the way for scalable screening solutions [2,3].

### 2.4. Future Implications for Population Screening and Prevention

Cancer screening at the population level coupled with early detection certainly is cost effective, as it not only lowers the overall morbidity and mortality from the disease but also delivers practical, worthwhile healthcare services. The future of cancer screening per se will be highly personalized, yet it may hinge on multi-cancer early detection (MCED) tests, which aim to identify multiple types of cancer from a single sample. The Galleri screening blood test [Galleri, available online: https://www.galleri.com/, accessed on 20 August 2025] is currently undergoing clinical trials. It is intended for adults with an elevated risk of cancer, such as those aged 50 and older, and is designed to detect over 50 cancer types through ctDNA analyses. It is available under CLIA certification, i.e., is accessible as a laboratory-developed test (LDT), though false positive and false negative results do occur. If the trials are successful and the data is compelling, MCED tests could transform population-wide screening programs, particularly for cancers like pancreatic or esophageal cancer, which lack effective early detection methods. By enabling earlier diagnosis and intervention, these tests could dramatically shift the focus of oncology toward economical prevention and proactive cancer care.

## 3. Biomarkers in Cancer Diagnosis and Prognosis

### 3.1. Their Current Use in Confirming a Diagnosis and Determining Prognosis

Biomarkers are vital for confirming cancer diagnoses, predicting disease progression, and tailoring therapeutics modalities. Generally, non-invasive procedures are preferred for sampling, and biofluids are favored as the specimens by far. Currently, confirmation techniques can be broadly classified as either imaging-based (CT, SPECT, MRI, and PET) or molecular-based (genes, mRNA, proteins, and peptides). Though biomarkers are pivotal, it is noteworthy to mention that only a few have gained approval from the FDA. Challenges remain with biomarkers for different cancer types due to the complexity of cancer development and significant variations among patients. For example, PSA in prostate cancer, HER2/ER/PR in breast cancer, CA-125 in ovarian cancer, AFP in liver cancer, and KRAS in colorectal cancer (CRC) are particularly important. To illustrate, the overexpression or amplification of the HER2 gene is characteristic of aggressive forms of breast cancer, i.e., it is a good predictor of diminished survival and reduced relapse. On the other hand, ER^+^ breast cancers are predictive of the response to endocrine therapy. In colorectal cancer, mutations in the KRAS gene (KRAS^+^) are associated with CRC resistance and worse patient outcomes (i.e., they may not respond as well to EGFR inhibitors). Overall, these biomarkers provide clinicians with essential information for staging the disease, assessing its likely course, and developing tailored treatment plans.

### 3.2. Advances in Diagnostic Biomarker Research

In spite of study design biases, potential technical artifacts, and the drawbacks associated with single-biomarker assessments, there are pragmatic reasons for their application and continued use in healthcare settings. However, there has been a realization that biomarker panels or profiling is more valuable in cancer testing and personalized management. There are both cancer-specific and pan-cancer panels that are commercially available, with the majority relying on next-generation sequencing (NGS) (genomic profiling), i.e., NGS-based tests [5]. Liquid biopsies as a mini-invasive sample collection method play a significant role in early detection, enabling molecular biomarker measurements. At the moment, most biomarker investigations are centered on the detection of CTCs, ctDNA, and EVs, specifically subtype variety exosomes. The development of immunotherapy has had a tremendous impact on cancer treatment. The discovery of the PD-1/PD-L1 interaction, which suppresses the immune system and allows for tumor immune evasion, has revolutionized the management of cancers like melanoma and non-small-cell lung cancer (NSCLC) [6]. An elevated PD-L1 expression significantly correlates with high-risk prognostic indicators and a decreased survival. In other words, high PD-L1 levels can indicate that patients are more likely to benefit from immune checkpoint inhibitors, such as Pembrolizumab^®^. However, PD-L1 is an insufficient predictor of the treatment outcomes, especially for patients with immune-impaired, inflammatory profiles, suggesting that anti-inflammatory agents should be included in personalizing immunotherapy for these hard-to-treat cancer patients [7].

### 3.3. Novel Approaches to Improving Accuracy and Specificity

A wide range of innovative biomolecular technologies are being pursued to improve the specificity and sensitivity of diagnostic biomarker assays—for instance, multi-omics approaches, nanotechnology techniques, multiplex immunoassays, electrochemical biosensors, and surface plasmon resonance (SPR), to name a few, including the standard imaging protocols/technological enhancements. Advances in NGS in the commercial realm have been highlighted above, as they provide comprehensive coverage and high sensitivity and specificity for detecting tumor mutations, fusions, and copy number alterations. Nanotechnology [8], for instance, uses engineered nanoparticles to bind specifically to cancer cells, enhancing either detection or therapy. Prominent examples include the FDA-approved Abraxane^®^ (albumin-bound Paclitaxel) and Doxil^®^ (liposomal doxorubicin). Advancements in clinical imaging tools and techniques such as molecular probes, contrast agents, and tracers are also refining the diagnostic accuracy, as they are designed to specifically target certain molecular features or physiological processes associated with cancer, like superparamagnetic iron oxide nanoparticle (SPION) contrast agents. Positron emission tomography (PET), when combined with biomarker-specific tracers, like the peptide radiotracer [^68^Ga]-NOTA-WL12 for the PD-L1 expression in NSCLC (the first peptide radiotracer to be applied in humans) or the ^18^F-labeled nanobody for HER2-positive breast cancers, are demonstrating success in cancer patients, joining [^68^Ga]-PSMA-11, which is already FDA-approved for prostate cancer.

### 3.4. Their Potential Impact on Treatment Planning and Patient Outcomes

The future of early cancer diagnosis and recurrence detection would likely involve integrating multi-omics profiles—combining genomic, proteomic, and metabolomic data—along with AI analyses to generate a detailed molecular tumor portrait for each patient [3]. When combined with novel, validated biomarkers, this would potentially impact prognosis, risk stratification, treatment planning, response monitoring, and thus ultimately patient outcomes. This holistic approach should lead to highly personalized therapy plans that target the specific drivers of a patient’s cancer, therefore minimizing ineffective therapies and the associated side effects, and facilitating an equally important reduction in healthcare costs. For the deadliest, hardest-to-treat cancers (i.e., those with the lowest survival rates), such as pancreatic, esophageal, liver, and brain (glioblastoma) cancers, this holistic approach could hopefully translate into improved survival rates and an improved quality of life, marking a significant step forward in precision oncology [5].

## 4. Biomarkers in Treatment Selection and Monitoring

### 4.1. Predictive Biomarkers for Selecting Targeted Therapies

Incorporating precision oncology into standard care has become a central priority within the healthcare agenda. This emphasis reflects not only its crucial role in improving patient outcomes but also its potential to optimize resource use by preserving healthcare capacity while deploying advanced technologies. Prognostic and predictive biomarker categorization is having a significant impact on the management of solid and hematologic cancers [9]. These biomarkers enable clinicians to move beyond a one-size-fits-all model, providing risk assessments or treatments tailored to the molecular profile of a patient’s tumor, thereby enhancing efficacy while minimizing unnecessary toxicity. Predictive biomarkers specifically inform the selection of the most beneficial therapies for individual patients, such as HER2 in breast cancer indicating Trastuzumab^®^/Pertuzumab^®^. In lung cancer (NSCLC), EGFR mutations and ALK gene rearrangement predict the responsiveness to tyrosine kinase inhibitors (TKIs) like Erlotinib^®^/Gefitinib^®^ and Crizotinib^®^, respectively. Meanwhile, BRCA1/2 mutations in breast and ovarian cancer patients, in particular platinum-sensitive ones, indicate eligibility for PARP inhibitors like Olaparib^®^ [2,9].

### 4.2. Monitoring Treatment Responses and Detecting Resistance

Providing insights into a tumor’s behavior and response to therapy is paramount to treatment success. Biomarkers enable treatment response monitoring and the identification of resistance. ctDNA and CTCs are powerful examples of biomarkers for treatment monitoring and minimal residual disease/resistance assessments [3]. Serial measurements (tracking changes in the levels) of ctDNA can be used to monitor the tumor burden over time, thus offering real-time insights into whether a therapy is working. For example, a decline in ctDNA levels may indicate a positive response, while a rise could signal resistance. Similarly, CTC analyses can identify androgen receptor splice variants (AR-Vs) in prostate cancer. In cases of resistance in NSCLC, biomarkers such as secondary EGFR mutations can guide the switch to alternative therapies from first-generation to second-generation inhibitors, such as Osimertinib^®^, ensuring that treatment remains effective throughout the disease course. Likewise, BRAF mutations in melanoma guide the use of BRAF and MEK inhibitors [3].

### 4.3. Emerging Biomarkers and Technologies for Precision Oncology

Precision oncology is being driven by the development of novel biomarkers and cutting-edge technology. As a case in point, tumor-educated platelets (TEPs) are emerging as a highly promising and potentially important new biomarker in the field of cancer research and diagnostics since different cancers induce distinct changes in TEP content (RNA and protein profiles). Another emergent biomarker is the tumor mutational burden (TMB), a measure of the number of mutations in a tumor, which is evolving as a predictor of the success of immunotherapy/better survivability, mainly in response to immune checkpoint inhibitors in cancers like melanoma, NSCLC, and colorectal and bladder cancers [2,6]. Other recognized cancer biomarker candidates include exosomes, miRNA, and lncRNA. Finally, epigenetic biomarkers are also gaining ground and becoming valuable in oncology, e.g., the hypermethylation of tumor suppressor genes like SEPT9 for the detection of colorectal cancer.

Moreover, the implementation of liquid-biopsy-based monitoring (as discussed above) and single-cell sequencing technologies offers granular insights into tumor heterogeneity, clonal evolution, and rare cell populations, which can inform mid-course treatment adjustments and predict relapses with higher precision. Taken together, these innovations are transforming treatment optimization from a reactive to a proactive and precision-guided paradigm, fundamentally reshaping precision cancer care.

### 4.4. Future Directions in Therapy Optimization

Cancer treatment optimization—The future of cancer therapy is shifting more toward dynamic, data-driven therapeutic strategies that adapt to each patient’s unique tumor biology in real time. At the forefront of this evolution are adaptive clinical trials leveraging ongoing biomarker feedback to modify the treatment plans during the course of patient care—for instance, the detection of early resistance, thus prompting a switch to alternative agents before clinical progression is evident [10]; the consideration of combination therapies, which is becoming more common; and/or metronomic chemotherapy [11], which is the continuous administration of chemotherapeutics at a lower dose without prolonged drug-free periods.

To illustrate, so far in 2025, the FDA has granted accelerated approval for several innovative lung cancer treatments, including Zongertinib^®^, another oral, irreversible TKI for HER2-mutant NSCLC after progression on chemo-immunotherapy; Sunvozertinib^®^, another oral, irreversible TKI for EGFR exon-20 insertion mutations after progression on chemotherapy; Datopotamab deruxtecan-dlnk (Datroway^®^), an antibody–drug conjugate for HR^+^/HER2^−^ breast cancer previously treated with endocrine-based therapy and chemotherapy and for EGFR-mutant NSCLC after progression on prior therapies; and Telisotuzumab vedotin-tllv (Emrelis ^®^), another antibody–drug conjugate for patients with a high c-MET expression after progression on prior therapies. These therapies represent meaningful advances, but the access to biomarker testing must expand in order for the benefits of these drugs to be seen.

Companion diagnostics—Tests that identify patients likely to benefit from specific drugs are becoming increasingly sophisticated, moving beyond single-marker assays to multiplex platforms that integrate multi-omics data input. These tests not only improve the treatment matching but also reduce unnecessary patient suffering, drug toxicity, and healthcare costs, henceforth aligning clinical practice with the principles of value-based oncology [2,6].

Artificial intelligence (AI) and machine learning (ML)—Algorithms are expected to tailor and optimize therapy further by analyzing vast datasets from electronic health records, imaging, and multi-omics platforms [12,13]. These computational tools can forecast therapeutic responses, detect subtle resistance patterns, and even suggest novel drug combinations tailored to an individual’s tumor profile. Furthermore, they could optimize clinical workflows for precision oncology patient care [13].

AI-designed cancer drugs are accelerating the discovery phase and are being carried forward into clinical trials, like ISM5939, an oral inhibitor of ENPP1 for solid tumors (IND-filed); an MTX-531 inhibitor with a dual-targeting mechanism (pan-PI3K/EGFR) for solid tumors as well, such as head and neck squamous cell carcinoma (HNSCC) and gastrointestinal tumors; and CDK12/13 inhibitors (transcription impairment), targeting a variety of cancers (gastric, ovarian, prostate, CRC, liver, etc.).

## 5. Challenges in the Clinical Translation of Cancer Biomarkers

### 5.1. Technical Challenges

Despite their potential, cancer biomarkers face technical hurdles that limit their clinical utility. Low sensitivity and specificity can undermine the reliability of tests, particularly in early-stage cancers, where biomarker levels—like those of ctDNA—are often low. Variability in the test results across platforms or laboratories complicates their adoption further, including ethnic disparities in biomarkers [2,5]. Overcoming these issues requires the development of more sensitive detection methods, such as improved liquid biopsy techniques and multiplexed or advanced amplification technologies.

### 5.2. Biological Variability and Tumor Heterogeneity

Cancer’s inherent heterogeneity poses a significant challenge. Potentially, biomarkers may vary between patients or even within different regions of the same tumor, leading to inconsistent results [2,5]. For example, a biopsy from one tumor site might detect a biomarker that is absent elsewhere, skewing the diagnosis or treatment decisions. Addressing this requires biomarkers that can capture the full spectrum of a tumor’s biology (biomarkers that are verified, validated, and qualified) or strategies for sampling multiple regions, such as through liquid biopsies, which reflect the systemic tumor dynamics [2].

### 5.3. Regulatory and Reimbursement Hurdles

The journey from discovery to clinical use is arduous and is typically impeded by regulatory and financial barriers [2,5,6]. Biomarker tests must undergo rigorous validation/qualification in large-scale clinical trials to gain approval from regulatory agencies like the FDA, a process that takes time and funding. Even after approval, securing reimbursement from insurance providers is challenging, especially for novel tests lacking long-term outcome data. These obstacles delay the integration of promising biomarkers into routine healthcare.

### 5.4. The Need for Large-Scale Validation and Standardization

To ensure their reliability, biomarkers must be validated in diverse populations through large-scale population studies that account for biological variability and possibly ethnicity [2,5]. Standardization of the testing protocols, laboratory instrumentation, operator variability, and interpretation criteria are also critical aspects of ensuring consistency across healthcare settings. Collaborative efforts among researchers, clinicians, and industry stakeholders are essential to conducting these studies and establishing global standards, hence accelerating the clinical adoption of biomarker-based tools.

## 6. Conclusions

Extraordinary strides in cancer biomarkers have been made in recent years, driven by innovations in multi-omics, like genomics and proteomics, and detection technologies. These advancements are already reshaping cancer care, enabling earlier population screening, timely detection, more accurate diagnoses, and, importantly, personalized treatment strategies that improve patient outcomes. However, challenges like technical limitations, tumor heterogeneity, and regulatory hurdles must be overcome to fully harness their potential.

Looking forward, multidimensional assessments of cancer biology, the integration of multi-omics data, AI-driven analytics, and real-time monitoring tools promise to revolutionize oncology and aid in decision-making. As novel biomarkers are uncovered, existing biomarkers are refined, and platform technologies are developed through research, our vision of precision oncology—where every patient receives tailored, effective care—draws closer. Achieving this future requires sustained investment in cancer research and cancer clinical care; interdisciplinary collaboration; a commitment to addressing translational challenges; and, last but not least, partnerships with regulatory agencies and government policy-makers.

In summary, cancer biomarkers, from their discovery to the realization of their clinical utility, are at the forefront of the healthcare evolution in oncology. Their continued development will not only enhance survival rates but also usher in a new era of holistic cancer precision care that is more proactive, precise, and patient-centered [14].

## Data Availability

This is an opinion, literature-based editorial article. No new data were created or analyzed in this study. Data sharing does not apply to this article.
